# Short-Term Outcomes of XEN45 Standalone versus Combined with Phacoemulsification in Open-Angle Glaucoma Patients: A Retrospective Study

**DOI:** 10.3390/jcm13010157

**Published:** 2023-12-27

**Authors:** Vittorio Pirani, Francesco Virgili, Vincenzo Ramovecchi

**Affiliations:** ASUR—Area Vasta 3, Ospedale di San Severino Marche, Via Del Glorioso, 8, 62027 San Severino Marche, Italy; vittorio.pirani@sanita.marche.it (V.P.); francesco.virgili@sanita.marche.it (F.V.)

**Keywords:** open-angle glaucoma, intraocular pressure, XEN implant, MIGS, needling, success

## Abstract

The XEN45 is a minimally invasive glaucoma surgery device commonly used in clinical practice. This retrospective study included consecutive patients with open-angle glaucoma who underwent a XEN45 implant with mitomycin C, either alone or in combination with phacoemulsification, between June 2015 and March 2021. The primary end point was the mean change in intraocular pressure (IOP) from the baseline to month 6. A total of 677 eyes, 395 (58.3%) in the XEN alone group and 282 (41.7%) in the XEN+Phaco group, were included in this study. The preoperative IOP was significantly lowered from 28.7 ± 8.6 mmHg and 25.4 ± 6.9 mmHg to 13.5 ± 5.0 mmHg and 13.5 ± 4.1 mmHg at month 6 in the XEN solo and XEN+Phaco groups, respectively, with *p* < 0.0001 each. The mean (95% confidence interval) number of ocular hypotensive medications was significantly reduced from 3.3 (3.2–3.4) to 0.2 (0.1–0.2) and from 3.1 (2.9–3.2) to 0.2 (0.1–0.2) in the XEN solo and XEN+Phaco groups, respectively, with *p* < 0.0001 each. Needling was performed in 228 (33.7%) eyes. Conclusions: the XEN implant significantly reduces both IOP and the number of ocular hypotensive medications. IOP lowering was higher in the XEN solo group than in the XEN+Phaco one, although such a difference was only evident during the first month after surgery.

## 1. Introduction

The XEN gel stent implant is a minimally invasive glaucoma surgery (MIGS) device, which was designed to provide a safer alternative to the traditional filtering glaucoma surgery while maintaining a good intraocular pressure (IOP)-lowering profile [1,2,3].

The XEN45 gel stent (Allergan, an Abbvie company, Irvine, CA, USA) allows a controlled aqueous humor flow from the anterior chamber to the subconjunctival space [1,2,3].

The XEN45 gel stent is usually delivered using an ab interno approach through a corneal incision [4,5,6,7,8,9,10,11,12,13,14,15]. However, as surgeons gain experience with the device, different changes, which aim to provide better clinical outcomes, have been introduced in the implantation technique [16,17,18].

Different studies have evaluated the efficacy and safety of XEN45 implants in clinical practice, showing their good effectiveness profile [6,7,8,9,10,11,12,13,14,15].

The aim of the current study was to evaluate the efficacy (in terms of the IOP-lowering effect) and safety of the XEN45 implant, either alone or in combination with phacoemulsification, in patients with open-angle glaucoma (OAG) over a period of 6 months in a real-world scenario.

## 2. Materials and Methods

Design: This is a retrospective and single-center study conducted on consecutive patients who underwent a XEN45 (Allergan, an Abbvie company, Irvine, CA, USA) implant, either alone or in combination with phacoemulsification, between June 2015 and March 2021.

This study adhered to the tenets of the Declaration of Helsinki, and all patients signed a written general consent to participate in the study, which was approved by the Ethics Committee of the Ospedale di San Severino Marche.

Participants: Consecutive patients, aged ≥18 years, with insufficiently medically controlled early-to-advanced OAG, according to Hodapp et al. [19]; intolerance to topical hypotensive treatments; or poor treatment adherence, who underwent a XEN45 implant, either alone or in combination with cataract surgery, were included in this study. Patients with uncontrolled ocular hypertension despite medical therapy were also included.

Patients with any form of glaucoma other than OAG were excluded from the analysis. Additionally, patients with any abnormality preventing reliable applanation tonometry in the study eye(s); progressive retinal or optic nerve disease due to any cause; or conjunctival scarring, which, in the surgeon’s opinion could compromise the procedure outcomes, were also excluded.

The patients were instructed to withdraw their topical and systemic ocular hypotensive medications on the day of surgery.

Surgical technique: All procedures were performed under topical anesthesia by the same experienced surgeon (VR). The XEN implant was placed in the superior nasal quadrant using a standard ab interno technique [4,12,13].

In short, the conjunctival upper-nasal quadrant was marked 3 mm from the limbus. Intraoperatively, 0.2 mL of mitomycin C (MMC) 0.01% was injected, under the tenon capsule, at 8 mm from the limbus. The pre-loaded injector needle was inserted, using an ab interno approach, at the inferotemporal quadrant by checking the position with the gonioscopy lens. The pre-loaded injector needle entered anteriorly into the trabecular meshwork and posteriorly into Schwalbe’s line; the implant was then injected. 

In those patients where cataract surgery was indicated, phacoemulsification was performed using the preferred surgical technique. After the intraocular lens was properly placed in the bag, XEN implantation followed, as previously indicated.

The standard postoperative care included antibiotic therapy 5 times a day for 1 week, and anti-inflammatory therapy with steroids 5 times daily for 1 month, which was slowly tapered over 12 weeks.

Study groups: The study sample was divided into two different groups: XEN solo (patients who underwent the XEN implant alone) and XEN+Phaco (patients who underwent combined XEN + phacoemulsification surgery).

Outcomes: The primary end point was the mean change in the IOP from the baseline to month 6.

The secondary end points included the mean IOP at month 6, the mean number of antiglaucoma medications and its changes from the baseline; the proportion of patients classified as a success; the proportion of patients achieving, at month 6, an IOP ≤ 21 mmHg, IOP ≤ 18 mmHg, IOP ≤ 16 mmHg, or ≤14 mmHg, irrespective of the % reduction; predictive factors associated with success; and the incidence of adverse events.

Definitions: Success was defined according to the World Glaucoma Association Consensus [20].

Complete success was defined as an IOP ≤ 21 mmHg and an IOP reduction ≥ 20%, without any hypotensive medication at month 6.

Qualified success was defined as an IOP ≤ 21 mmHg and an IOP reduction ≥ 20%, with/without topical hypotensive medication at month 6.

Patients with an IOP < 4 mmHg for more than two consecutive visits, those who needed further glaucoma surgery, or those who had surgery for complications were also considered a failure.

Statistical analysis: A standard statistical analysis was performed using MedCalc^®^ Statistical Software version 20.2 (MedCalc Software Ltd., Ostend, Belgium; 2022). 

Descriptive statistics of number (percentage), mean (standard deviation (SD)), mean (95% confidence interval (95% CI)), mean (standard error (SE)), median (interquartile range), or median (95% CI) were used, as appropriate. 

Data were tested for normal distribution using a D’Agostino–Pearson test.

The statistical analysis was similar to that reported by Monja-Alarcon et al. [21].

The repeated measures ANOVA test or Friedman’s two-way analysis test, as appropriate, was used to assess the changes in IOP and in the number of antiglaucoma medications. Post hoc analysis for pairwise comparisons was carried out with Scheffé’s method (ANOVA) or the Conover method (Friedman). The Mann–Whitney U test was used in the evaluation of the changes in IOP and in the number of ocular hypotensive medications between XEN solo and XEN+Phaco study groups.

Success survival rates were plotted for XEN solo and XEN+Phaco groups using Kaplan–Meier analysis and were compared using a log-rank test.

The repeated analysis of covariance (MANCOVA) was performed to assess the changes in IOP between study groups. The model included “type of surgery” (XEN alone or XEN+Phaco) as a factor and age, preoperative IOP, number of preoperative ocular hypotensive medications, previous glaucoma surgery, type of previous glaucoma surgery, and operated eye as covariates. 

A logistic regression model was used to estimate and test factors for their association with success. A backward strategy was adopted, with a statistically significant cut-off for variable screening of 0.05. Factors associated with success in the univariate analysis with *p* values < 0.1 were included in the multivariate analysis.

Categorical variables were compared using a chi-square test and Fisher’s exact test, as needed. A *p* value of less than 0.05 was considered significant.

## 3. Results

Six-hundred and seventy-seven eyes fulfilled the inclusion and exclusion criteria. A total of 395 (58.3%) eyes underwent XEN-alone surgery, and 282 (41.7%) eyes underwent XEN+Phaco. Mean age was 73.7 ± 10.7 years, and 224 eyes had undergone a previous glaucoma procedure (either laser or surgery). Table 1 shows the main clinical and demographic clinical characteristics of the study sample.

The mean preoperative IOP was significantly greater in the XEN solo group than in the XEN+Phaco group (Hodges–Lehmann median difference: 3.0 mmHg; 95% confidence interval: 2.0 to 4.0, *p* < 0.0001) (Table 1).

In the overall study sample, mean (95% CI) IOP was significantly lowered from 27.3 (26.7 to 27.9) mmHg at the baseline to 7.4 (6.9 to 7.8) mmHg; 10.3 (9.8 to 10.8) mmHg; 12.9 (12.4 to 13.4) mmHg; 13.5 (13.1 to 13.9) mmHg; 13.2 (12.9 to 13.5) mmHg; and 13.3 (13.1 to 13.4) mmHg at day 1, week 1, months 1, 2, 3, and 6 visits, respectively (repeated measures ANOVA and Greenhouse–Geisser correction; *p* < 0.0001 each, respectively). 

**Figure 1:**
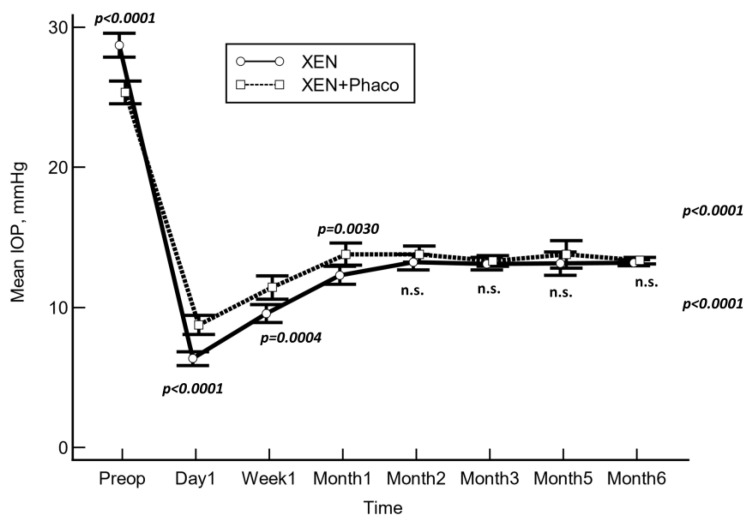
Mean intraocular pressure (IOP) over the course of follow-up in the XEN solo and XEN+Phaco groups. The vertical bars represent the 95% confidence interval. Mean IOP was significantly lower in the XEN solo group at day 1, week 1, and month 1, but no significant differences were observed at any of the other IOP time points measured (statistical significance was determined using the one-way ANOVA test with Scheffé’s method). n.s.: Not significant.

The mean preoperative IOP was significantly lowered from 28.7 ± 8.6 mmHg and 25.4 ± 6.9 mmHg to 13.5 ± 5.0 mmHg and 13.5 ± 4.1 mmHg at month 6 in the XEN solo and XEN+Phaco groups, respectively (*p* < 0.0001 each, repeated measures ANOVA (Figure 1)).

As compared to the baseline, the mean IOP was significantly lowered at every time point measured (*p* < 0.001 each, repeated measures ANOVA and Greenhouse–Geisser correction) in both study groups.

As compared to preoperative values, the mean (95% CI) IOP lowering was −15.2 (−16.3 to −14.3) mmHg and −11.8 (−12.8 to −10.9) mmHg in the XEN solo and the XEN+Phaco groups, respectively (*p* < 0.0001).

After adjusting for “age”, “preoperative IOP”, “number of preoperative ocular hypotensive medications”, and “operated eye”, the mean IOP lowering was greater in the XEN solo group than in the XEN+Phaco one at day 1, week 1, and month 1. However, no significant differences were observed at any of the remaining IOP time points measured (Table 2).

Unadjusted IOP lowering percentage was significantly greater in the XEN solo group (mean: 49.2%; 95% CI: 46.9% to 51.5%) than in the XEN+Phaco one (43.3%; 95% CI: 40.3% to 45.8%; *p* = 0.0008). However, after adjusting by “age”, “preoperative IOP”, “number of preoperative ocular hypotensive medications”, and “operated eye”, there were no significant differences between both groups (mean difference 0.7%, 95% CI: −2.3% to 3.6%, *p* = 0.6523), with IOP lowering significantly depending on “preoperative IOP” (*p* < 0.001).

The mean (95% CI) number of antiglaucoma medications was significantly reduced from 3.3 (3.2 to 3.4) to 0.2 (0.1 to 0.2) and from 3.1 (2.9 to 3.2) to 0.2 (0.1 to 0.2) in the XEN solo and XEN+Phaco groups (*p* < 0.0001 each), respectively.

At month 6, 358 (90.6%) eyes were classified as a success in the XEN solo group, with 323 (81.8%) eyes classified as a complete success. In the XEN+Phaco group, 243 (86.2%) eyes were classified as a success, with 222 (78.8%) eyes classified as a complete success. The success rates were similar in both groups (*p* = 0.0835). 

Table 3 shows the proportion of eyes that achieved different IOP targets irrespective of the percentage reduction from the baseline.

**Figure 2:**
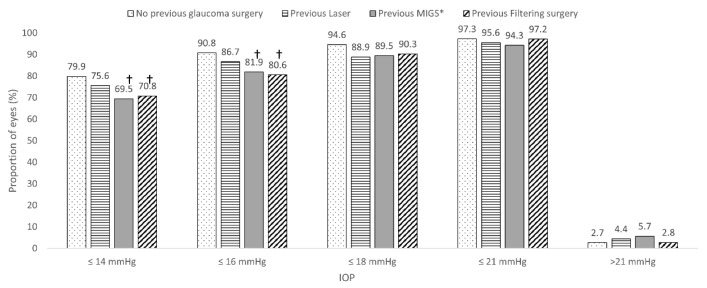
Overview of the impact of previous glaucoma surgical procedure (laser and/or filtering surgery) on the probability of achieving a specific target intraocular pressure. * Minimally invasive glaucoma procedures other than XEN. † *p* < 0.05 as compared to no previous glaucoma surgery. MIGS: Minimally invasive glaucoma surgery; IOP: Intraocular pressure.

As compared to those eyes that did not undergo previous glaucoma surgery, the proportion of eyes achieving a final IOP ≤ 14 mmHg and ≤16 mmHg was significantly lower in the eyes that underwent a previous MIGS device procedure and previous filtering surgery (Figure 2).

**Figure 3:**
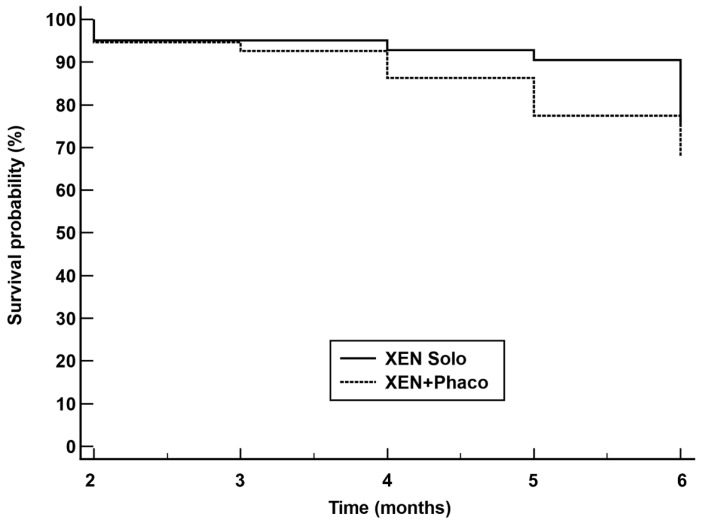
Kaplan–Meier survival curves for failure in eyes treated with XEN alone (XEN solo) (solid line) and combined surgery XEN + phacoemulsification (XEN+Phaco) (dotted line). Failure occurred in 37 (9.4%) XEN solo-treated eyes and 39 (13.8%) XEN+PHACO-treated eyes. Mean hazard ratio (HR) 0.62; 95% confidence interval 0.38 to 0.99; *p* = 0.0459.

The Kaplan–Maier survival analysis found that survival probability was higher in the XEN solo group than in the XEN+Phaco one (*p* = 0.0459) (Figure 3).

The logistic regression analysis found that “preoperative IOP”, “IOP lowering at day-1” and “IOP lowering at week-1”, and “have undergone a previous glaucoma surgery” were significantly associated with success in the univariate analysis (*p* < 0.0001, *p* < 0.0001, *p* < 0.0001, and *p* = 0.0274, respectively), but only the preoperative IOP was significantly associated with success in the multivariate analysis (*p* = 0.0054) (Table 4).

Needling was performed in 228 (33.7%) eyes. The XEN solo and the XEN+Phaco groups showed a similar proportion of needling procedures (32.2% versus 35.8%, *p* = 0.3290). Median time to the first needling procedure was lower in the XEN+Phaco group (42 days) than in the XEN solo one (59 days), although this difference was not statistically significant (*p* = 0.1336).

Regarding safety, 29 eyes had at least one postoperative adverse event along with 24 (6.1%) and 5 (1.8%) eyes in the XEN solo and the XEN+Phaco groups, respectively (Table 5).

## 4. Discussion

According to the results of the current study, the XEN45 stent, either alone or in combination with phacoemulsification, significantly lowered the IOP and reduced the number of ocular hypotensive medications in patients with OAG in a real clinical setting. 

Interestingly, this study suggested that in the short term, the IOP-lowering effect of the XEN45 device implanted alone was higher than that observed in patients undergoing the XEN implant combined with phacoemulsification, although the IOP-lowering effect was equal from month 1.

Additionally, the high proportion of patients achieving low target IOPs should be highlighted, with 74.3% and 85.1% of patients achieving an IOP ≤ 14 mmHg and ≤16 mmHg without treatment, respectively. There were no significant differences in the proportion of eyes that achieved a specific target IOP without treatment between the XEN and the XEN+Phaco groups, although the proportion of eyes achieving an IOP ≤ 14 mmHg and ≤16 mmHg with treatment was slightly but significantly greater in the XEN solo group (*p* = 0.0355 and *p* = 0.0467, respectively).

From a clinical point of view, different studies have reported the mid- and long-term efficacy, in terms of IOP lowering and the extent of ocular hypotensive medication reduction and safety of the XEN45 implant, either alone or in combination with phacoemulsification surgery, in OAG patients [6,7,8,9,10,11,12,13,14,15]. 

Regarding IOP lowering and reduction in glaucoma medication, the results of our study did not significantly differ from the currently available scientific evidence [6,7,8,9,10,11,12,13,14,15].

There are few studies evaluating the efficacy of the XEN implant in patients who have undergone previous glaucoma laser/surgical procedures [5,6,10].

Grover et al. [5], in a multi-center study that included advanced glaucoma (14.3% of patients) and refractory glaucoma (84.6% of patients), reported a success rate of 76.3%. 

Hengerer et al. [6] evaluated 242 eyes, of which 173 (70.2%) eyes had undergone a previous intervention (53 eyes (21.9%) a previous iStent implant and 52 eyes (21.5%) a previous trabeculectomy). They observed that complete success was achieved by 55.4% of eyes and qualified success by 73% of eyes [6]. 

These figures seem to be slightly lower than that observed in our study (88.8%), although their median follow-up was slightly longer (12 months) than ours (6 months). Additionally, success criteria were different.

As compared to eyes that did not undergo previous glaucoma surgery (79.9% and 90.8%, respectively), the proportion of eyes achieving a month 6 IOP ≤ 14 mmHg and ≤16 mmHg was significantly lower in the eyes that underwent a previous MIGS (69.5% and 81.9%, *p* < 0.05 each, respectively) procedure and those that underwent previous filtering surgery (70.8% and 80.6, *p* < 0.05 each, respectively). Nevertheless, the proportion of eyes achieving lower IOPs was relatively high.

The finding that the XEN 45 implant may be successfully used in patients with previous glaucoma procedures is interesting because, up to now, trabeculectomy with wound modulator agents and the glaucoma drainage devices is the treatment of choice in these patients. However, despite its good IOP-lowering effectiveness, this method is associated with a relatively high incidence of both early and late postoperative interventions and complications [22,23,24]. 

Many papers have evaluated the efficacy of the XEN45 device in a combined procedure with cataract surgery. However, there are conflicting reports in the literature regarding the superiority of the solo procedure over the combined procedure with cataract surgery [6,9,10,25,26]. 

Hengerer et al. [6] did not find significant differences in success rates between the XEN implanted alone and the combined procedure (XEN+Phaco). Similarly, Marcos-Parra et al. [9] and Karimi et al. [10] did not find significant differences in IOP lowering between XEN alone and the XEN+Phaco groups. However, Mansouri et al. [25] and Gillman et al. [26] found higher success rates in the XEN alone group. 

In our study, after adjusting by different covariates, there were no significant differences in IOP lowering between the XEN and the XEN+Phaco groups beyond the month 1. The adjusted IOP reduction rate at month 6 was not significantly different (mean difference 0.7%, 95% CI: −2.3% to 3.6%, *p* = 0.6523), and IOP lowering significantly depended on “preoperative IOP” (*p* < 0.001). Moreover, success rates were similar in both groups.

Considering the results of the current study, the XEN45 device in combination with cataract surgery does not impair the effectiveness of the procedure, although in the short term the IOP-lowering effect was greater with the XEN alone implantation, due probably to the higher preoperative IOP values.

However, according to Kaplan–Meier survival analysis, the XEN alone procedure reduces the relative risk of failure by 38% compared with the XEN+Phaco combined surgery.

The current study found that “preoperative IOP”, the “IOP lowering at day-1” and “IOP lowering at week-1”, and “have undergone a previous glaucoma surgery” were significantly associated with success in the univariate analysis, although only “preoperative IOP” was identified as predictor factor in the multivariate analysis.

When a relative IOP reduction is involved in the definition of success, higher baseline IOPs may be a predictor of success. In fact, if we consider a definition involving only a fixed threshold, irrespective of the percentage reduction from the baseline; for example, IOP ≤ 18 mmHg in preoperative IOP was not a predictive factor (odds ratio: 1.01; 95% CI: 0.98 to 1.04; *p* = 0.5894).

As regards the safety profile, the incidence and type of complications were similar to those previously published [6,7,8,9,10,11,12,13,14,15,25,26]. Two-hundred and twenty-eight (33.7%) eyes underwent a needling procedure. Additionally, three (1.7%) eyes required a new XEN implant.

In ophthalmology, current elastography techniques are able to assess biomechanical properties of different ocular tissues, such as the cornea or the sclera, and detect changes in biomechanical properties associated with changes in IOP [27,28]. Better knowledge of the impact of these anterior segment structures on different ocular disorders, such as glaucoma, will help clinicians to understand the relationship between corneal and scleral biomechanical properties and IOP [27,28,29].

Several limitations should be taken into consideration when interpreting the results of the current study. The first one is its retrospective design. Selection bias and potential confounders are inherent to retrospective studies. Nevertheless, the selection of strict inclusion/exclusion criteria, as well as the inclusion of many eyes, may have minimized these issues. 

Because we have analyzed surgeries carried out over almost 6 years, it should be noted that our clinical criteria for implanting the XEN device have varied over time. As our knowledge about the device expanded, the criteria for selecting patients have been refined to implant the device in the best candidates and thus obtain the best clinical outcomes. This fact may represent a bias, since, at the beginning, we included patients with unfavorable conditions, such as very high IOP, previous failure in filtering surgeries, or impaired conjunctiva. However, this fact also represents a strength of the study, since it accurately reflects the conditions of real clinical practice. The third limitation is the short follow-up. Glaucoma is a long-lasting disease, so we must establish the long-term effectiveness of its treatments. Although this study evaluated the effectiveness of XEN in the short term, we have evidence showing its good safety and efficacy profile in the long-term [11,26]. Additionally, baseline differences between groups might limit the validity of study conclusions. Nevertheless, it is essential to take into consideration that IOP lowering was adjusted by different covariates, which clearly minimizes that issue. Finally, the current study did not include a control group, which limits the ability to draw definitive conclusions about the efficacy of the XEN45 compared to other treatments.

## 5. Conclusions

In conclusion, the results of this study certainly suggested that the XEN implant, either alone or in combination with phacoemulsification, is an effective treatment for lowering IOP and reducing the need for ocular hypotensive medication while maintaining a good safety profile. We found greater IOP reductions in the XEN solo group than in the XEN+Phaco group. However, they were only evident during the first month after surgery. Additionally, we did not find any difference in either the success rates or the probability of achieving a specific target IOP. Additionally, among eyes that had undergone a previous glaucoma filtering procedure, the current study found a high success rate (83.3%), which opens the door for using the XEN45 device in these types of patients. Finally, the preoperative IOP was identified as an independent risk factor for success in this large cohort of patients, although it cannot be ruled out that this is due to how success was defined. 

The question of whether MIGS devices might replace conventional surgery in patients with previous glaucoma procedures remains debatable. Further studies, particularly those evaluating the effectiveness of these devices in patients with refractory glaucoma or their cost-effectiveness, are needed.

## Figures and Tables

**Table 1:** Overview of the main demographic and clinical characteristics of the study population.

	Overall (*n* = 677)	XEN Solo (*n* = 395)	XEN+Phaco (*n* = 282)	*p* ^a^
Age, years				0.1665
Mean (SD)	70.7 (10.7)	72.9 (11.8)	74.8 (8.7)	
95% CI	72.9 to 74.5	71.7 to 74.1	73.8 to 75.8	
Sex, *n* (%)				0.0012 ^b^
Women	289 (43.0)	148 (37.2)	141 (50.4)	
Men	383 (57.0)	244 (62.2)	139 (49.6)	
Eye, *n* (%)				0.5849 ^b^
Right	351 (52.2)	208 (53.1)	143 (50.9)	
Left	322 (47.8)	184 (46.9)	138 (849.1)	
BCVA, logMAR				<0.0001
Mean (SD)	0.48 (0.28)	0.54 (0.30)	0.39 (0.23)	
95% CI	0.46 to 0.50	0.51 to 0.57	0.37 to 0.42	
IOP, mmHg				<0.0001
Mean (SD)	27.3 (8.2)	28.7 (8.6)	25.3 (7.1)	
95% CI	226.7 to 28.9	27.9 to 29.6	24.4 to 26.1	
NOHM *				0.0026
Mean (SD)	3.2 (1.0)	3.3 (1.0)	3.1 (1.0)	
95% CI	3.1 to 3.3	3.2 to 3.4	2.9 to 3.2	
Mean defect, dB				0.0287
Mean (SD)	−15.1 (9.4)	−16.1 (9.2)	−13.9 (9.6)	
95% CI	−16.1 to −14.1	−17.4 to −14.8	−15.4 to −12.3	
Previous surgery ^1^, *n* (%)				<0.0001
Yes	408 (60.3)	298 (75.4)	110 (39.0)	
No	269 839.7)	97 (24.6)	172 (61.0)	
Previous glaucoma procedures ^2^, *n* (%)				<0.0001 ^b^
Yes	224 (54.9)	144 (48.3)	80 (72.7)	
No	184 (45.2)	154 (51.7)	30 (27.3)	
Previous glaucoma procedures ^2^, *n* (%)				0.5490 ^c^
Laser	46 (20.5)	27 (18.8)	19 (23.8)	
MIGS	105 46.9)	67 (46.5)	38 (47.5)	
Trabeculectomy	73 (32.6)	50 (34.7)	23 (28.8)	
Valve implant ^3^	0 (0.0)	0 (0.0)	0 (0.0)	

* Active principles (for example, fixed combinations count depending on the number of active principles). ^1^ Any type (including glaucoma procedures). ^2^ Any type (including laser, traditional filtering surgery, and minimally invasive glaucoma procedures other than XEN). ^3^ Includes Ahmed valve and Molteno implant. ^a^ Mann–Whitney test. ^b^ Fisher exact test. ^c^ Chi-squared test. SD: Standard deviation; CI: Confidence interval; BCVA: Best corrected visual acuity; IOP: Intraocular pressure; NOHM. Number of ocular hypotensive medications; MIGS: Minimally invasive glaucoma surgery.

**Table 2:** Mean changes in intraocular pressure (IOP) in eyes that underwent XEN solo (XEN) and those that underwent combined surgery (XEN+Phaco).

Mean Change in IOP, mmHg	XEN		XEN+Phaco		Difference		
	Mean	SE	Mean	SE	Mean (SE)	95% CI	*p* ^a^
Day 1	−21.1	0.3	−18.4	0.3	−2.7(0.4)	−3.5 to −1.8	<0.0001
Week 1	−18.0	0.5	−15.7	0.4	−2.3 (0.5)	−3.4 to −1.3	<0.0001
Month 1	−15.2	0.3	−13.3	0.4	−1.9 (0.5)	−2.9 to −0.9	0.0004
Month 3	−14.3	0.2	−13.9	0.2	−0.4 (0.3)	−1.0 to 0.3	0.3086
Month 6	−14.2	0.1	−14.0	0.1	−0.2 (0.2)	−0.6 to 0.2	0.2783

^a^ Repeated measures analysis of covariance (MANCOVA). The model included “Type of surgery” (XEN solo versus XEN+Phaco) as a factor and age, preoperative IOP, number of preoperative ocular hypotensive medications, previous glaucoma surgery, type of previous glaucoma surgery, and operated eye (right versus left) as covariates. IOP = Intraocular pressure; SE = Standard error; CI = Confidence interval.

**Table 3:** Overview of the proportion of patients who achieved specific intraocular pressure levels, with and without hypotensive medication, at month 6 visit.

	Without Treatment				With Treatment			
	Overall	XEN Solo	XEN+Phaco	*p*	Overall	XEN Solo	XEN+Phaco	*p*
≤14 mmHg, *n* (%)	454 (74.3)	272 (76.4)	182 (71.4)	0.1634	44 (66.7)	30 (76.9)	14 (51.9)	0.0355
≤16 mmHg, *n* (%)	520 (85.1)	301 (84.6)	215 (84.3)	0.9196	52 (78.8)	34 (87.2)	18 (66.7)	0.0467
≤18 mmHg, *n* (%)	550 (90.0)	317 (89.0)	233 (91.4)	0.3299	57 (86.4)	35 (89.7)	22 (81.5)	0.3438
≤21 mmHg, *n* (%)	582 (95.3)	337 (94.7)	245 (96.1)	0.4212	61 (92.4)	37 (94.9)	24 (88.9)	0.3681
>21 mmHg, *n* (%)	29 (4.7)	19 (5.3)	10 (3.9)	0.4212	5 (7.6)	2 (5.1)	3 (11.1)	0.3681

**Table 4:** Univariate and multivariate analysis of the 677 eyes included in the study to evaluate the potential factors for success. Factors associated with success in the univariate analysis at *p* < 0.1 were included in the multivariate analysis.

	Success ^1^			
	Univariate		Multivariate	
Variable	Odds Ratio (95% CI)	*p*	Odds Ratio (95% CI)	*p*
Age (per year)	1.01 (0.99 to 1.03)	0.3965	N.A.	N.A.
Preoperative treatment (per active principle added)	0.84 (0.66 to 1.08)	0.1696	N.A.	N.A.
Preoperative IOP (per mmHg higher)	1.12 (1.08 to 1.17)	<0.0001	1.09 (1.03 to 1.16)	0.0054
IOP lowering at day 1 (per mmHg greater)	1.07 (1.04 to 1.10)	<0.0001	1.02 (0.97 to 1.07)	0.4635
IOP lowering at week 1 (per mmHg greater)	1.06 (1.03 to 1.08)	<0.0001	1.01 (0.98 to 1.05)	0.5072
IOP lowering at month 1 (per mmHg greater)	1.02 (0.99 to 1.05)	0.1094	N.A.	N.A.
Eye (reference RE)	0.76 (0.47 to 1.23)	0.2593	N.A.	N.A.
Sex (reference woman)	1.08 (0.67 to 1.75)	0.7463	N.A.	N.A.
Previous glaucoma surgery (reference No.)	0.61 (0.37 to 0.99)	0.0437	0.66 (0.38 to 1.04)	0.0947
Type of previous glaucoma surgery (reference No.)				
Laser	0.88 (0.42 to 1.88)	0.7487	N.A.	N.A.
MIGS *	1.20 (0.59 to 2.45)	0.6178	N.A.	N.A.
Trabeculectomy	0.82 (0.33 to 2.03)	0.6693	N.A.	N.A.
Type of surgery (reference XEN alone)	0.64 (0.40 to 1.04)	0.0714	0.78 (0.40 to 1.51)	0.4636

^1^ IOP ≤ 21 mmHg and an IOP reduction ≥ 20%, with or without any hypotensive medication at the last follow-up visit. * Other than XEN. N.A.: Not applicable.

**Table 5:** Overview of the postoperative complications in the eyes that underwent XEN alone (XEN solo) or XEN + phacoemulsification (XEN+Phaco).

	Type of Surgery		
Complication	XEN	XEN+Phaco	Overall
Choroidal detachment, *n* (%)	8 (2.0)	1 (0.35)	9 (1.3)
Conjunctival cysts, *n* (%)	0 (0.0)	1 (0.35)	1 (0.15)
Extrusion, *n* (%)	2 (0.5)	1 (0.35)	3 (0.44)
Flat anterior chamber, *n* (%)	1 (0.025)	0 (0.0)	1 (0.15)
Hemovitreous, *n* (%)	1 (0.025	0 (0.0)	1 (0.15)
Hypertension, *n* (%)	1 (0.025)	2 (0.70)	3 (0.44)
Hypotony, *n* (%)	2 (0.5)	0 (0.0)	2 (0.30)
Malignant glaucoma, *n* (%)	3 (0.75)	0 (0.0)	3 (0.44)
XEN (new implant), *n* (%)	3 (0.75)	0 (0.0)	3 (0.44)
XEN malposition, *n* (%)	1 (0.025)	0 (0.0)	1 (0.15)
Endophthalmitis, *n* (%)	1 (0.025)	0 (0.0)	1 (0.15)
Vitreoretinal surgery, *n* (%)	1 (0.025)	0 (0.0)	1 (0.15)
Total *	24	5	29

* Eight eyes had two complications, and three eyes had three complications.

## Data Availability

The data that support the findings of this study are available from the corresponding author (V.R.) upon reasonable request.

## References

[1] Lavia C., Dallorto L., Maule M., Ceccarelli M., Fea A.M. (2017). Minimally-invasive glaucoma surgeries (MIGS) for open angle glaucoma: A systematic review and meta-analysis. PLoS ONE.

[2] Pillunat L.E., Erb C., Jünemann A.G., Kimmich F. (2017). Micro-invasive glaucoma surgery (MIGS): A review of surgical procedures using stents. Clin. Ophthalmol..

[3] Shah M. (2019). Micro-invasive glaucoma surgery—An interventional glaucoma revolution. Eye Vis..

[4] Laborda-Guirao T., Cubero-Parra J.M., Hidalgo-Torres A. (2020). Efficacy and safety of XEN 45 gel stent alone or in combination with phacoemulsification in advanced open angle glaucoma patients: 1-year retrospective study. Int. J. Ophthalmol..

[5] Grover D.S., Flynn W.J., Bashford K.P., Lewis R.A., Duh Y.J., Nangia R.S., Niksch B. (2017). Performance and Safety of a New Ab Interno Gelatin Stent in Refractory Glaucoma at 12 Months. Am. J. Ophthalmol..

[6] Hengerer F.H., Kohnen T., Mueller M., Conrad-Hengerer I. (2017). Ab Interno Gel Implant for the Treatment of Glaucoma Patients with or without Prior Glaucoma Surgery: 1-Year Results. J. Glaucoma.

[7] Schlenker M.B., Gulamhusein H., Conrad-Hengerer I., Somers A., Lenzhofer M., Stalmans I., Reitsamer H., Hengerer F.H., Ahmed I.I.K. (2017). Efficacy, Safety, and Risk Factors for Failure of Standalone Ab Interno Gelatin Microstent Implantation versus Standalone Trabeculectomy. Ophthalmology.

[8] Reitsamer H., Sng C., Vera V., Lenzhofer M., Barton K., Stalmans I., Apex Study Group (2019). Two-year results of a multicenter study of the ab interno gelatin implant in medically uncontrolled primary open-angle glaucoma. Graefes Arch. Clin. Exp. Ophthalmol..

[9] Marcos-Parra M.T., Salinas-López J.A., López-Grau N.S., Ceausescu A.M., Pérez-Santonja J.J. (2019). XEN implant device versus trabeculectomy, either alone or in combination with phacoemulsification, in open-angle glaucoma patients. Graefes Arch. Clin. Exp. Ophthalmol..

[10] Karimi A., Lindfield D., Turnbull A., Dimitriou C., Bhatia B., Radwan M., Gouws P., Hanifudin A., Amerasinghe N., Jacob A. (2019). A multi-centre interventional case series of 259 ab-interno Xen gel implants for glaucoma, with and without combined cataract surgery. Eye.

[11] Lenzhofer M., Kersten-Gomez I., Sheybani A., Gulamhusein H., Strohmaier C., Hohensinn M., Burkhard Dick H., Hitzl W., Eisenkopf L., Sedarous F. (2019). Four-year results of a minimally invasive transscleral glaucoma gel stent implantation in a prospective multi-centre study. Clin. Exp. Ophthalmol..

[12] Ibáñez-Muñoz A., Soto-Biforcos V.S., Rodríguez-Vicente L., Ortega-Renedo I., Chacón-González M., Rúa-Galisteo O., Arrieta-Los Santos A., Lizuain-Abadía M.E., Del Río Mayor J.L. (2020). XEN implant in primary and secondary open-angle glaucoma: A12-month retrospective study. Eur. J. Ophthalmol..

[13] Fea A.M., Bron A.M., Economou M.A., Laffi G., Martini E., Figus M., Oddone F. (2020). European study of the efficacy of a cross-linked gel stent for the treatment of glaucoma. J. Cataract Refract. Surg..

[14] Wagner F.M., Schuster A.K., Emmerich J., Chronopoulos P., Hoffmann E.M. (2020). Efficacy and safety of XEN®-Implantation v. trabeculectomy: Data of a “real-world” setting. PLoS ONE.

[15] Theilig T., Rehak M., Busch C., Bormann C., Schargus M., Unterlauft J.D. (2020). Comparing the efficacy of trabeculectomy and XEN gel microstent implantation for the treatment of primary open-angle glaucoma: A retrospective monocentric comparative cohort study. Sci. Rep..

[16] Panarelli J.F., Yan D.B., Francis B., Craven E.R. (2020). XEN Gel Stent Open Conjunctiva Technique: A Practical Approach Paper. Adv. Ther..

[17] Vera V., Gagne S., Myers J.S., Ahmed I.I.K. (2020). Surgical Approaches for Implanting Xen Gel Stent without Conjunctival Dissection. Clin. Ophthalmol..

[18] Tan N.E., Tracer N., Terraciano A., Parikh H.A., Panarelli J.F., Radcliffe N.M. (2021). Comparison of Safety and Efficacy between Ab Interno and Ab Externo Approaches to XEN Gel Stent Placement. Clin. Ophthalmol..

[19] Hodapp E., Parrish R., Anderson D. (1993). Clinical Decisions in Glaucoma.

[20] Heuer D.K., Barton K., Grehn F., Shaarawy T., Sherwood M., Shaarawy T.M., Sherwood M.B., Grehn F. (2008). Consensus on definitions of success. Guidelines on Design and Reporting of Surgical Trials.

[21] Monja-Alarcón N., Perucho-Martínez S., Buenasmañanas-Maeso M., Toledano-Fernández N. (2022). Does mitomycin-C concentration have any influence on XEN45 gel stent outcomes in a real-world setting?. Graefes Arch. Clin. Exp. Ophthalmol..

[22] Gedde S.J., Schiffman J.C., Feuer W.J., Herndon L.W., Brandt J.D., Budenz D.L., Tube versus Trabeculectomy Study Group (2012). Treatment outcomes in the Tube Versus Trabeculectomy (TVT) study after five years of follow-up. Am. J. Ophthalmol..

[23] Jampel H.D., Musch D.C., Gillespie B.W., Lichter P.R., Wright M.M., Guire K.E., Collaborative Initial Glaucoma Treatment Study Group (2005). Perioperative complications of trabeculectomy in the collaborative initial glaucoma treatment study (CIGTS). Am. J. Ophthalmol..

[24] Gedde S.J., Feuer W.J., Shi W., Lim K.S., Barton K., Goyal S., Ahmed I.I.K., Brandt J., Primary Tube Versus Trabeculectomy Study Group (2018). Treatment Outcomes in the Primary Tube Versus Trabeculectomy Study after 1 Year of Follow-Up. Ophthalmology.

[25] Mansouri K., Guidotti J., Rao H.L., Ouabas A., D’Alessandro E., Roy S., Mermoud A. (2018). Prospective Evaluation of Standalone XEN Gel Implant and Combined Phacoemulsification-XEN Gel Implant Surgery: 1-Year Results. J. Glaucoma.

[26] Gillmann K., Bravetti G.E., Rao H.L., Mermoud A., Mansouri K. (2021). Combined and stand-alone XEN 45 gel stent implantation: 3-year outcomes and success predictors. Acta Ophthalmol..

[27] Sun M.G., Son T., Crutison J., Guaiquil V., Lin S., Nammar L., Klatt D., Yao X., Rosenblatt M.I., Royston T.J. (2022). Optical coherence elastography for assessing the influence of intraocular pressure on elastic wave dispersion in the cornea. J. Mech. Behav. Biomed. Mater..

[28] Li R., Qian X., Gong C., Zhang J., Liu Y., Xu B., Humayun M.S., Zhou Q. (2023). Simultaneous Assessment of the Whole Eye Biomechanics Using Ultrasonic Elastography. IEEE Trans. Biomed. Eng..

[29] Zhang J., Murgoitio-Esandi J., Qian X., Li R., Gong C., Nankali A., Hao L., Xu B.Y., Shung K.K., Oberai A. (2022). High-Frequency Ultrasound Elastography to Assess the Nonlinear Elastic Properties of the Cornea and Ciliary Body. IEEE Trans. Ultrason. Ferroelectr. Freq. Control.

